# NiS_2_@MoS_2_ Nanospheres Anchored on Reduced Graphene Oxide: A Novel Ternary Heterostructure with Enhanced Electromagnetic Absorption Property

**DOI:** 10.3390/nano9020292

**Published:** 2019-02-19

**Authors:** Zhi Zhang, Xuliang Lv, Yiwang Chen, Pin Zhang, Mingxu Sui, Hui Liu, Xiaodong Sun

**Affiliations:** Key Laboratory of Science and Technology on Electromagnetic Environmental Effects and Electro-optical Engineering, The Army Engineering University, Nanjing 210007, China; zhangnjjn@163.com (Z.Z.); xllu1957@126.com (X.L.); chenyw1357@163.com (Y.C.); zhangpnj01@163.com (P.Z.); plasmx@126.com (M.S.); liuhh1005@163.com (H.L.)

**Keywords:** graphene, core–shell, heterostructure, electromagnetic absorption

## Abstract

For the purposes of strength, military equipment camouflage, and protecting the health of organisms, electromagnetic wave absorbing materials have received a lot of attention and are widely studied. In addition to having a strong absorption intensity and a wide effective absorption bandwidth, materials that are lightweight, thermally stable, and antioxidative are also highly desirable. In this study, we fabricated core–shell structured NiS_2_@MoS_2_ nanospheres anchored on reduced graphene oxide (rGO) nanosheets (NiS_2_@MoS_2_/rGO) by a simple two-step hydrothermal method. The combination ratio was adjusted to achieve proper impedance matching. The electromagnetic parameters and the absorption performance were investigated in detail. A composite loaded with 30 wt.% of the sample achieved a minimum reflection loss (RL) value of −29.75 dB and the effective bandwidth (RL value of less than −10 dB) ranged from 4.95 GHz to 18.00 GHz (13.05 GHz), with a thickness ranging from 1.5 mm to 4.0 mm. This study proved that the generated significant interfacial polarization and synergetic interaction between components can result in NiS_2_@MoS_2_/rGO composites with enhanced electromagnetic absorption performance.

## 1. Introduction

With large-scale applications of electromagnetic (EM) equipment in various fields, EM interference and EM pollution have become serious issues [1,2,3,4,5]. In general, electromagnetic wave absorbing material is a functional material; it can attenuate EM waves effectively, convert EM energy into thermal energy, or make EM waves dissipate through interference [2,6,7]. Therefore, EM wave absorbing materials have drawn much attention. To satisfy the increasing demand, EM wave absorbing materials with a wide absorption band, strong absorption performance, high stability, and antioxidation properties are urgently needed [8,9,10]. Traditional absorbers, such as metallic materials, have strong absorption performances due to their dielectric constant, but such drawbacks as high density and high cost restrict their further application [11]. Nanomaterials play a wide-ranging role in electronics, photovoltaics, biomaterials, energy storage, and EM wave absorption due to multiple merits, such as their special morphology and low density [12]. To date, great efforts have been made to design and synthesize nanomaterials that have strong EM wave absorbing performances [13] and diverse structures, such as nanorods, nanosheets, nanospheres, nanocubes, etc.

Reduced graphene oxide (rGO), which has a combination of advantages, such as high permittivity, high dielectric loss, remarkable thermal and mechanical properties, as well as low density, has attracted great interest in the field of EM wave absorbing materials [14]. However, rGO can hardly meet the requirement of applicability because of its high conductivity [15]. Excessive conductivity leads to an unsatisfactory impedance matching, so EM waves reflect strongly on the surface of the absorber [16]. The reflection loss (RL) of EM wave absorbers for application is at least −10 dB, while the value of pure rGO barely reaches −7 dB. To solve this problem, it is necessary to select some low dielectric constant materials to composite with rGO, as this will increase the interfacial polarization of the composite and adjust the impedance matching to achieve a satisfactory absorptive effect. Song et al. reported a 3D rGO–ZnO composite with the highest reflection loss of −27.8 dB and an absorption bandwidth of 4.2 GHz (below 10 dB with a thickness of 4.8 mm) [17]. Qu et al. synthesized Fe_3_O_4_ composited with graphene in a 3D structure. They found a maximum absorption of −27 dB at 8.0 GHz and a frequency range of 6.5–10.3 GHz [18]. Although these materials revealed good EM wave absorption performance, the narrow effective absorption frequency bandwidth became an inevitable defect that limited the further application of such materials. To solve this problem, a lot of research has focused on composing an appropriate nanostructure anchored on graphene-based composites. Wang et al. reviewed the electromagnetic interference shielding based on rGO and graphene. The preparation methods of carbon nanostructures and their nanocomposites are reviewed. The electromagnetic absorption properties of carbon nanocomposites synthesized by different methods are evaluated objectively and systematically [19]. Zhang et al. reported a graphene/thorns-like polyaniline/α-Fe_2_O_3_@SiO_2_ composite with the highest reflection loss of −50.06 dB and absorption bandwidth of 14.4 GHz (below 10 dB with a thickness of 2.3 mm) [20]. Sun and co-workers synthesized laminated magnetic graphene (rGO–Fe_3_O_4_) using a simple solvothermal route. They reported that the as-prepared composite had the highest reflection loss of −26.4 dB at 5.3 GHz with a coating layer thickness of 4.0 mm [21]. The results of the studies presented in this section have demonstrated that such rGO-based nanocomposites possess an enhanced EM absorption performance.

As is well known, nanomaterials with core–shell and york–shell structures have high interfacial polarization due to the synergistic effects of both the cores and shells [8,22,23,24,25]. Therefore, a number of studies have explored nanomaterials with core–shell and york–shell structures to achieve high EM wave absorbing performances [26,27]. Hossein et al. fabricated core–shell PPy/MnFe_2_O_4_ and found that the microwave absorbing property had an enhanced magnetic loss at 8–12 GHz. The composite exhibited a minimum reflection loss of −12 dB at 11.3 GHz with a thickness of 1.5 mm [28]. Yin et al. synthesized core–shell structured C/ZnO nanoparticles, achieving a maximum absorption at 11 GHz of −52.0 dB with a sample thickness of 1.75 mm [29]. However, non-ferromagnetic core–shell structured composites cannot satisfy the practical application standard because of the single absorption mechanism [30]. It is necessary to design new core–shell structural composites, with dielectric materials acting as shells and magnetic materials as cores, anchored on graphene for the sake of exerting a dielectric loss and magnetic loss, as well as achieving a great EM wave absorption effect [31].

Herein, we describe the fabrication of core–shell structured NiS_2_@MoS_2_ nanospheres anchored on rGO nanosheets (NiS_2_@MoS_2_/rGO) by a simple two-step hydrothermal method. In this work, MoS_2_ acted as the shell by forming the electric polarization center of the composite; MoS_2_ contacted rGO to produce interface polarization and adjust the dielectric constant of the composite appropriately. At the same time, NiS_2_ acted as the core, giving rise to magnetic loss, contacting MoS_2_ to produce another interface polarization, obtaining a good impedance match. We altered the combination ratio to adjust the impedance match of the NiS_2_@MoS_2_/rGO composites. In the end, the EM absorption performance of the as-prepared samples was investigated, and the possible absorption mechanisms were analyzed in detail. On the basis of numerous measurements, it was concluded that this NiS_2_@MoS_2_/rGO absorber is characterized by a good EM wave absorbing performance with a wide absorbing frequency, strong absorption, good compatibility, and low density.

## 2. Experimental Section

### 2.1. Materials

Most of the chemical reagents used in this work were purchased from Aladdin Chemical Co. Ltd (Shanghai, China), including nickel chloride hexahydrate (NiCl_2_·6H_2_O), ethylene glycol (EG), ammonium molybdate ((NH_4_)_6_Mo_7_O_24_·2H_2_O), and thiocarbamide (CH_4_N_2_S). The GO powder was provided by Xianfeng Chemical Co. Ltd (XF002-2, Nanjing, China). The NiS_2_@MoS_2_/rGO composites were synthesized through a two-step method.

### 2.2. Preparation of NiS_2_ Nanospheres

The NiS_2_ microspheres were synthesized via a simple hydrothermal method, as reported previously [32]. To produce the A solution, 0.9506 g of nickel chloride hexahydrate (NiCl_2_·6H_2_O) was well-dispersed in 50 mL of ethylene glycol (EG). For the B solution, 0.213 g of sulfur powder was dispersed in 50 mL of ethylene glycol (EG) under sonication for 10 min until the solution became clear. Then, the B solution was added to the A solution under magnetic stirring for 30 min at room temperature. Subsequently, 1.6 g of polyvinyl pyrrolidone (PVP-30,000), which had been dispersed in EG (50 mL), was added stepwise to the hybrid solution (A and B). The mixture was stirred vigorously for another 30 min and transferred to a Teflon-lined, stainless-steel autoclave (200 mL capacity). After reacting at 200 °C for 12 h in the autoclave, the solution was cooled to room temperature. Finally, the final precipitates were rinsed with distilled water and absolute ethanol and dried in vacuum at 50 °C for 15 h for further synthesis.

### 2.3. Synthesis of NiS_2_@MoS_2_/rGO Composites

The NiS_2_@MoS_2_/rGO composites were synthesized using a hydrothermal method. Firstly, 0.05 g of GO powder was added to 50 mL of distilled water under sonication for 60 min. Then, a certain amount of ammonium molybdate ((NH_4_)_6_Mo_7_O_24_·2H_2_O) was dispersed in 20 mL of distilled water and stepwise injected into the GO solution. After magnetic stirring for 20 min at room temperature, a certain amount of thiocarbamide, which was dispersed in 20 mL of distilled water, was added dropwise to the above solution and stirring was maintained for another 20 min at room temperature. Afterward, a certain amount of as-prepared NiS_2_ nanospheres, which were prepared in the previous step, was added. The mixture solution continued to be magnetically stirred for 60 min at 70 °C, after which it was transferred to a Teflon-lined, stainless-steel autoclave (200 mL) and reacted at 200 ℃ for 12 h. During this reaction, the pressure facilitated the hydrothermal reduction of GO [33]. Finally, the black composites were centrifuged, rinsed with distilled water and absolute ethanol several times, and dried in a vacuum at 50 °C for 15 h. For convenience, the NiS_2_@MoS_2_/rGO composites synthesized under different reagent ratios are denoted as sample A, sample B, and sample C. The detailed experimental reagent ratios are listed in Table 1.

### 2.4. Characterization and Measurement

The crystal structure of the samples was determined using a Bruker D8 Advanced X-ray diffractometer (XRD). The XRD patterns with Cu K_α_ radiation (λ = 1.5406 Å) at 40 kV and 40 mA were recorded in the range of 2θ = 5–80°. Field emission scanning electron microscopy (FE-SEM, Hitachi S4800) was used to determine the microstructure of the composite. Further characterization of the NiS_2_@MoS_2_/rGO composites was performed using transmission electron microscopy with energy dispersive X-ray spectroscopy (TEM/EDS, JEL-2100F, JEOL, Tokyo, Japan) and a high-resolution transmission electron microscope (HRTEM, Tecnai G^2^ F20, FEI company, Hillsboro, America). X-ray photon spectroscopy (XPS) was performed on an XPS spectrometer (ESCALAB 250, Thermo, America).

### 2.5. EM Absorption Measurement

The measured samples used for EM absorption measurements were prepared by compounding NiS_2_@MoS_2_/rGO composites with different proportions of wax (20 wt.%, 30 wt.%, 40 wt.%) and fabricating the corresponding cylindrical shaped compacts (Φ_out_ = 7.00 mm, Φ_in_ = 3.04 mm). Then, the microwave scattering parameters were recorded at 2–18 GHz with a coaxial wire method using a vector network analyzer (Agilent N5224A PNA). The vector network analyzer (VNA) works by using a signal generator to scan the sample in a frequency band. Four scattering parameters (S11, S22, S12, and S21) of the two-port network were measured respectively and the electromagnetic parameters of the sample were derived based on the measured scattering parameters. The VNA used in this experiment is a two-port measurement, which usually has built-in grounding devices, without the need for additional earthing devices. In the measuring device, the angle of the incident electromagnetic wave is 90°.

## 3. Results and Discussion

### 3.1. Characterization of Samples

**Figure 1:**
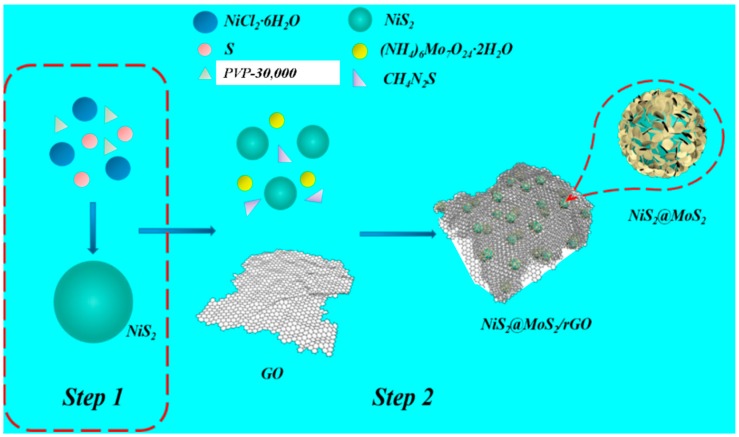
Schematic illustration of the formation process of the NiS2 nanospheres and NiS_2_@MoS_2_/rGO composites.

The synthesis processes of the NiS_2_ microspheres and NiS_2_@MoS_2_/rGO composites are schematically depicted in Figure 1. Firstly, the NiS_2_ nanospheres were synthesized via a simple hydrothermal method. Secondly, the NiS_2_ nanospheres were added to the mixed solution of MoS_2_ and GO, which were stirred well. Thirdly, the mixed solution was maintained with magnetic stirring for 60 min at 70 °C and then synthesized through a hydrothermal method at 200 °C for 12 h. During this process, the NiS_2_ nanospheres and MoS_2_ nanoplates formed core–shell structured NiS_2_@MoS_2_ nanospheres due to homologous molecular affinity and GO was transformed into rGO via a redox reaction. At the same time, the NiS_2_@MoS_2_ nanospheres were anchored onto reduced graphene oxide nanosheets.

**Figure 2:**
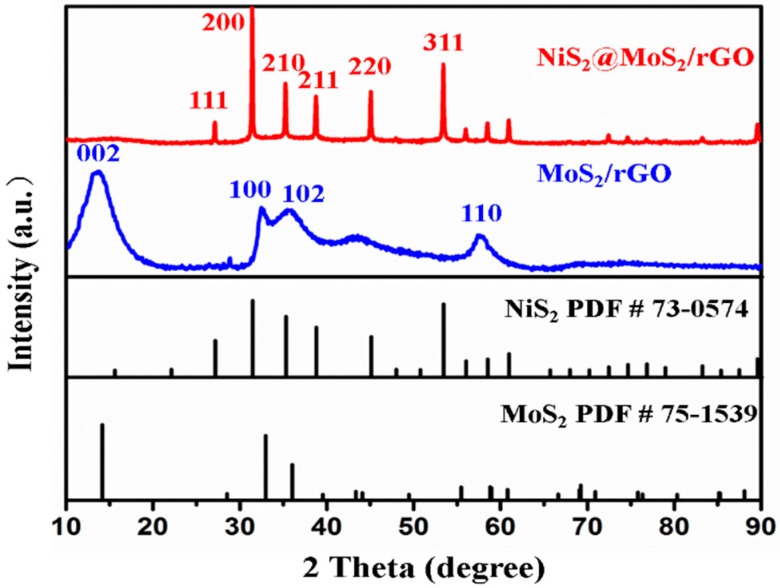
XRD patterns of MoS_2_/rGO and NiS_2_@MoS_2_/rGO composites.

The phase identification and phase purity of the NiS_2_@MoS_2_/rGO composites were investigated by the XRD technique. The XRD spectrum of Sample B is shown in Figure 2 and the XRD spectrum of MoS_2_/rGO. The PDF card of NiS_2_ (73-0574), and the PDF card of MoS_2_ (75-1539) are shown for comparison. When compared with PDF 73-0574, the presence of NiS_2_ can be clearly confirmed. It can be seen that most of the diffraction peaks correspond with the NiS_2_ PDF; only a few of the peaks are weaker due to the effect of MoS_2_ and rGO. Through the comparison with the PDF of MoS_2_, we found that the XRD spectrum of MoS_2_/rGO matches the PDF of MoS_2_ (PDF#75-1539) well. Four characteristic diffraction peaks at around 2θ = 14.1°, 32.9°, 37.1°, and 58.7° corresponding to the crystal faces (002), (100), (102), and (110), respectively, can be assigned to hexagonal MoS_2_. Due to the low crystallinity of rGO, it is difficult to see the characteristic diffraction peak from C (002) in the spectrum. However, it is difficult to find the diffraction peaks of MoS_2_ and rGO in the XRD spectrum of NiS_2_@MoS_2_/rGO. This is because the crystallinity of MoS_2_ and rGO is far weaker than that of NiS_2_ and the diffraction peaks of NiS_2_ and MoS_2_ partly overlap [34].

**Figure 3:**
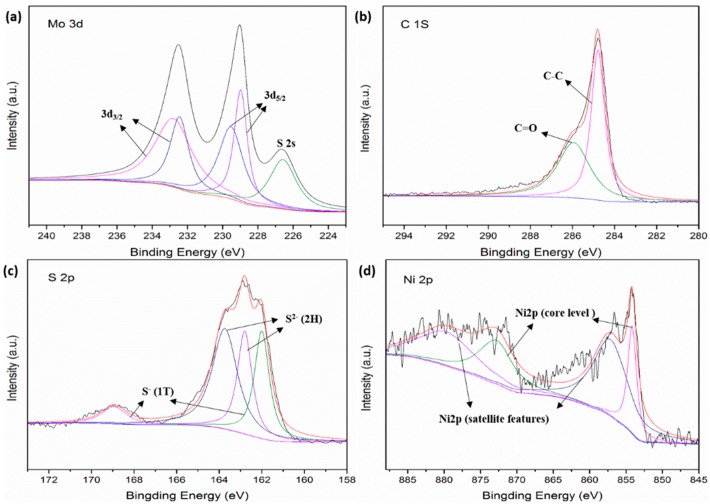
XPS spectra of (**a**) Mo 3d, (**b**) C 1s, (**c**) S 2p, and (**d**) Ni 2p.

In order to study the chemical composition of NiS_2_@MoS_2_/rGO more thoroughly, the XPS patterns are provided in Figure 3. Mo 3d spectra are presented in Figure 3a, in which the two peaks at 229.55 eV and 229.0 eV are attributed to Mo 3d_5/2_ and the two peaks at 232.85 eV and 232.45 eV are assigned to Mo 3d_3/2_ [35]. The peak at 226.6 is due to the existence of a few S species [36]. The peaks of C 1s in Figure 3b located at 284.8 eV and 285.95 eV correspond to the C–C bonds of aromatic rings and C=O bonds of carbonyl, respectively. As shown in Figure 3d, Ni 2p consists of four main binding energy peaks [15]. The peaks at 854.2 eV and 873.0 eV are attributed to the core level of Ni 2p. The other two binding energy peaks are also assigned and correspond to the satellite features of Ni 2p at 857.35 eV and 880.8 eV [37]. From the S 2p spectrum (Figure 3c), it is easy to find that the S atom exists in NiS_2_@MoS_2_/rGO composites with two different phases (metallic 1T-phase and semiconducting 2H-phase). The peaks at 162.0 eV and 168.95 eV are attributed to the metallic 1T-phase NiS_2_@MoS_2_, while those at 162.8 eV and 163.75 eV correspond to the semiconducting 2H-phase NiS_2_@MoS_2_.

**Figure 4:**
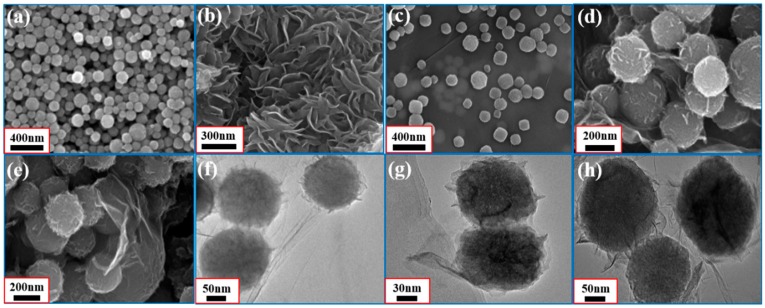
SEM images of (**a**) pure NiS_2_, (**b**) MoS_2_; SEM images of (**c**) sample A, (**d**) sample B, (**e**) sample C; TEM images of (**f**) sample A, (**g**) sample B, (**h**) sample C.

SEM and TEM images are presented in Figure 4 to characterize the microstructure and the morphologies of the materials in this study. Figure 4a,b show the pure NiS_2_ nanospheres and the pure flower-like MoS_2_ with sheet structures, respectively. The average diameter of NiS_2_ nanospheres is about 150 nm. The SEM images of rGO are shown in Figure S1. As revealed by the SEM images (Figure 4c,d), the average diameter of the core–shell NiS_2_@MoS_2_ is about 200 nm, and that of sample C (Figure 4e) is about 230 nm due to the increased MoS_2_ content. Figure 4f–h show TEM images of different component proportions of the NiS_2_@MoS_2_/rGO composites. The core–shell structure of NiS_2_@MoS_2_ can be observed clearly on the surface of rGO (Figure 4f). The inset of Figure 4g exhibits the way in which the core–shell structured NiS_2_@MoS_2_ nanospheres are anchored on the rGO nanosheets. It can be clearly observed that the MoS_2_ shells act as a perfect link between rGO and NiS_2_ to form a complete composite structure. Figure 4h shows that the NiS_2_ nanospheres are wrapped by the MoS_2_ nanosheets, resulting in a core–shell structure. The thickness of the MoS_2_ shell is about 15–45 nm due to the different component proportions of MoS_2_. Moreover, the sizes of the NiS_2_ cores remained constant after the hydrothermal process.

**Figure 5:**
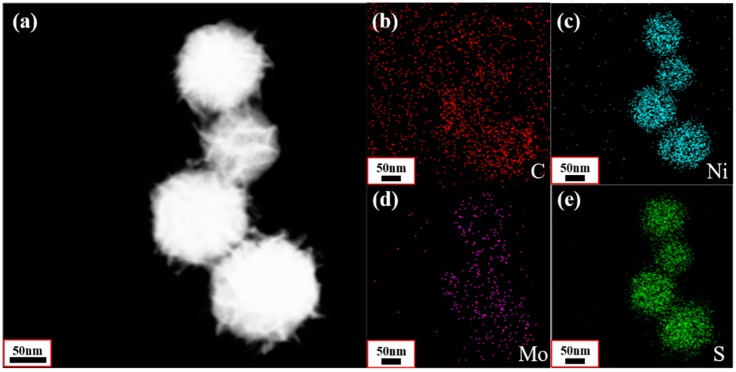
(**a**) TEM image and (**b–e**) EDS mapping images of NiS_2_@MoS_2_/rGO composites.

**Figure 6:**
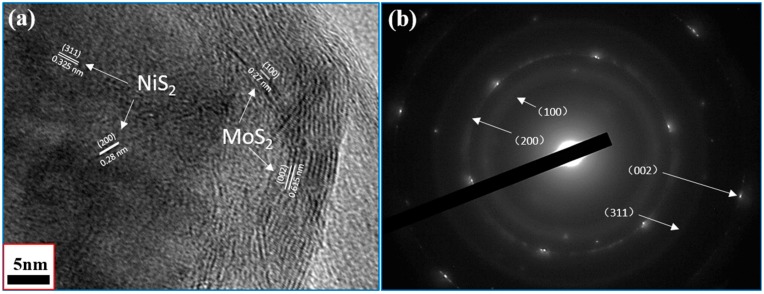
(**a**) HRTEM images and (**b**) SAED pattern of NiS_2_@MoS_2_/rGO composites.

To visually demonstrate the distribution of elements in the NiS_2_@MoS_2_/rGO composites, energy dispersive X-ray spectrometry (EDS) (Figure S2) and element maps of C, Ni, Mo, and S (Figure 5) are displayed. In Figure 5b, the C element in red has a uniform distribution, which indicates that rGO acts as the base layer of the composites. Meanwhile, the Ni, Mo, and S elements are found on the core–shell structure, demonstrating that the NiS_2_ nanospheres (Ni and S elements) are evenly coated by MoS_2_ nanosheets. In order to validate the core–shell structure of NiS_2_@MoS_2_ in detail, a HRTEM image and the selected area electron diffraction (SAED) pattern of the NiS_2_@MoS_2_/rGO composites are presented in Figure 6. As shown in Figure 6a, the lattice planes with a d spacing of 0.28 nm and 0.325 nm are attributed to the (200) and (311) crystal planes of the NiS_2_ core and 0.27 nm and 0.615 nm are attributed to the (100) and (002) crystal planes of the MoS_2_ shell. In Figure 6b, the SAED pattern reveals four diffraction rings: Two of them correspond to the (200) and (311) crystal planes of the NiS_2_ core and the others correspond to the (100) and (002) crystal planes of the MoS_2_ shell. Although the characteristics of rGO are not identified in either the HRTEM or SAED patterns due to the weak scattering power of rGO, the XPS, EDS, and element mapping results provide evidence of the presence of rGO.

### 3.2. Electromagnetic Absorption Property

**Figure 7:**
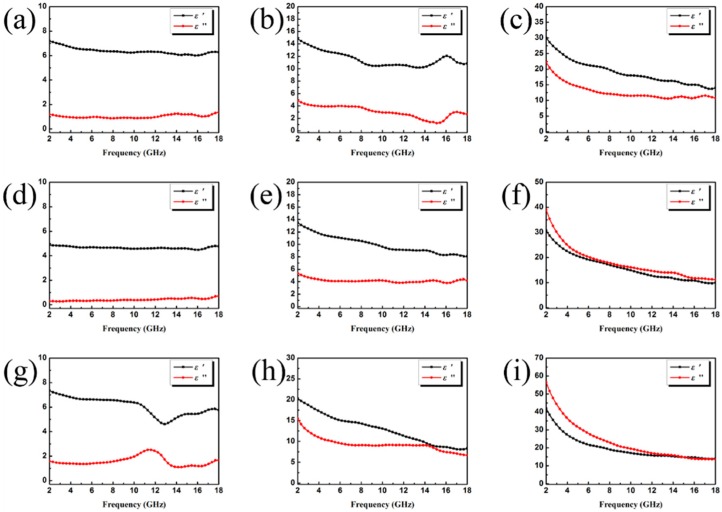
Frequency dependence of the real and imaginary parts of complex permittivity of sample A with a filler loading of (**a**) 20 wt.%, (**b**) 30 wt.%, (**c**) 40 wt.%; sample B with a filler loading of (**d**) 20 wt.%, (**e**) 30 wt.%, (**f**) 40 wt.%; sample C with a filler loading of (**g**) 20 wt.%, (**h**) 30 wt.%, (**i**) 40 wt.%.

**Figure 8:**
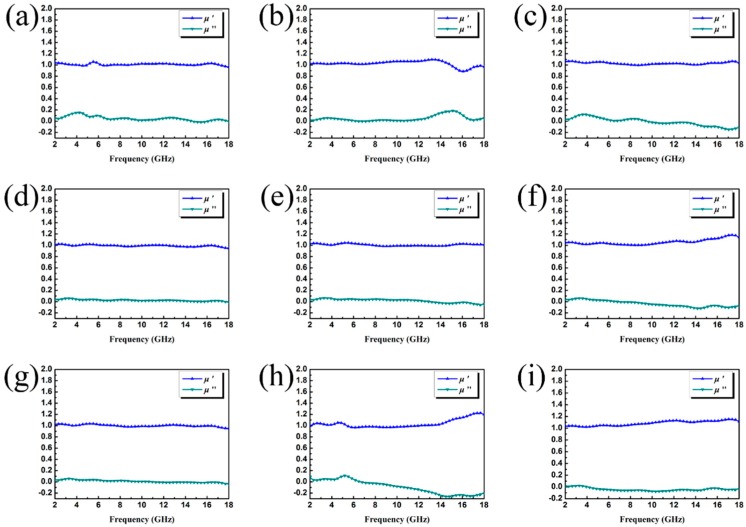
Frequency dependence of real and imaginary parts of complex permeability of sample A with a filler loading of (**a**) 20 wt.%, (**b**) 30 wt.%, (**c**) 40 wt.%; sample B with a filler loading of (**d**) 20 wt.%, (**e**) 30 wt.%, (**f**) 40 wt.%; sample C with a filler loading of (**g**) 20 wt.%, (**h**) 30 wt.%, (**i**) 40 wt.%.

The EM attributes (μ_r_ and ε_r_) of the as-prepared samples loaded with different fillers were measured, where μ_r_ is the complex permeability, μ_r_ = μ′ − jμ″; ε_r_ is the complex permittivity, ε_r_ = ε′ − jε″; the results are shown in Figure 7 and Figure 8. In general, ε′ and μ′ account for the amount of polarization in the samples and represent the ability to store electric and magnetic energy, while ε′′ and μ′′ denote the dissipated electric and magnetic energies. Note that ε′ and ε′′ increase upon increasing the filler loading, indicating a strong dielectric loss. However, the relatively high permittivity is not beneficial to the impedance match and could result in more reflection on the surface. Because the dielectric polarization failed to approach the alternating EM field in the high-frequency range, the values of the real part of the permittivity show a decreasing trend upon increasing the frequency within 2–18 GHz, generally. The curves in Figure 7b and Figure 7g present a complicated frequency-dependent behavior. Note that in the frequency ranges of 15–17 GHz (Figure 7b) and 10–14 GHz (Figure 7g), we find strong resonance peaks, which are related to high conductivity, electronic spin, skin effects, and charge polarization due to the polarized centers and point effects. The permeability curves of all samples exhibit similar values in the whole testing frequency range. For the μ′ and μ′’ value (Figure 8a,b,h), the obvious resonance peaks can be attributed to the natural resonance. Furthermore, the negative value could be due to the motion of charges in an EM field.

The electromagnetic absorption (EA) performance of the composites can be evaluated by the values of reflection loss (RL). A smaller RL suggests highly efficient absorption. The tested frequency range is from 2 to 18 GHz (λ = c/f decreases from 150 to 17 mm); thus, this measurement process can be considered under far-field conditions because the source-to-shield distance is much larger than the free-space wavelength [38]. According to the transmission line theory, the RL at the interface can be expressed using the following equations:

(1) $$Z_{in}=Z_{0}\sqrt{\frac{\mu_{r}}{\varepsilon_{r}}}\tan(h)(j\frac{2\pi fd}{c}\sqrt{\varepsilon_{r}\mu_{r}}),$$

(2) $$RL(\mathrm{dB})=20\mathrm{lg}\left|\frac{z_{in}-z_{0}}{z_{in}+z_{0}}\right|,$$

where ƒ is the frequency, d is the thickness, and c is the velocity of light in the free space. Z_0_ is the free space of impedance matching. When the RL is lower than −10, −20, or −30 dB, it means more than 90%, 99%, or 99.9% of the EM energy is absorbed, respectively. It has been acknowledged that a bandwidth lower than −10 dB can be regarded as an effective EA bandwidth.

**Figure 9:**
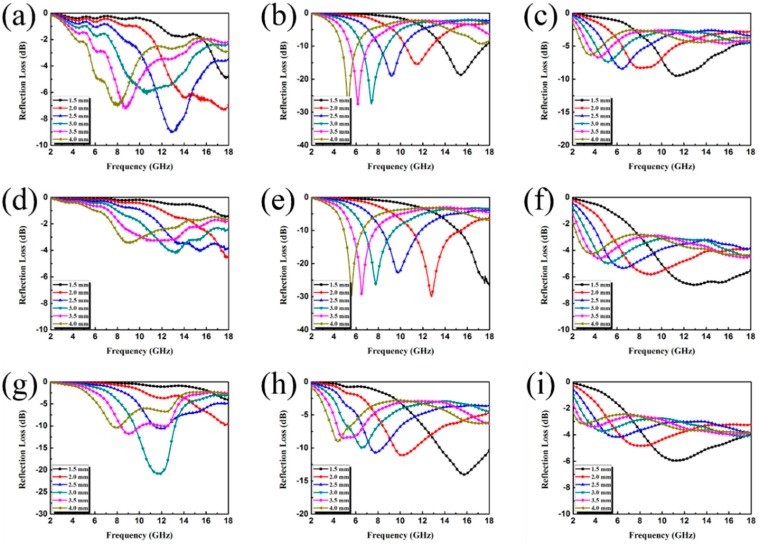
Reflection loss curves of the filler loadings of (**a,b,c**) sample A; (**d,e,f**) sample B; (**g,h,i**) sample C, with 20 wt.%, 30 wt.%, and 40 wt.% in wax composites with thicknesses from 1.5 to 4.0 mm in the frequency range of 2–18 GHz.

**Figure 10:**
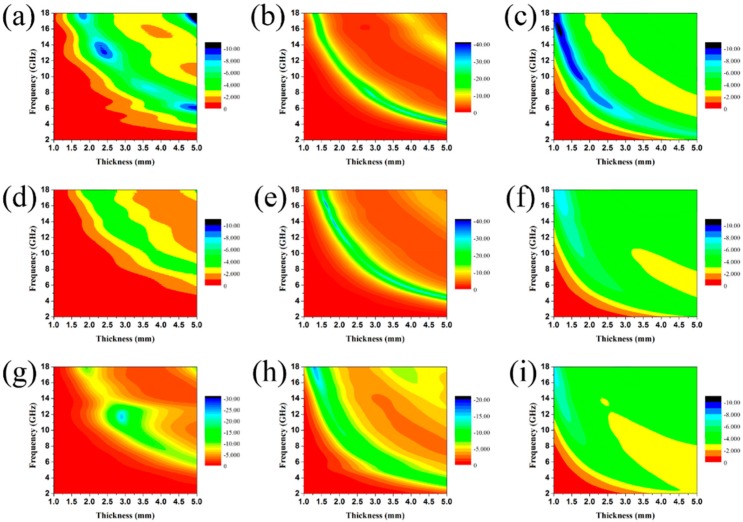
Reflection loss contour maps of the filler loadings of (**a,b,c**) sample A; (**d,e,f**) sample B; (**g,h,i**) sample C, with 20 wt.%, 30 wt.%, and 40 wt.% in wax composites with thicknesses from 1.0 to 5.0 mm in the frequency range of 2–18 GHz.

The thickness-dependent EA performance of the three as-fabricated composite samples was investigated and the results are shown in Figure 9 and Figure 10. In Figure 9f, we find that high filler loading leads to poor EA performance. This is because the extremely high values of ε′ and ε′′ cause an impedance mismatch and thus lead to EM waves reflecting on the surface rather than being absorbed. Comparing sample A and sample C, it is clear that the composite loaded with 30 wt.% of sample B has the best EA performance for each thickness. The minimum RL value is −29.75 dB when the thickness is 4.0 mm and a broad effective bandwidth (RL value of less than −10 dB) can cover from 4.95 GHz to 18.00 GHz (13.05 GHz) with a thickness ranging from 1.5 mm to 4.0 mm. It can be concluded from the results that the EA properties of the as-prepared materials are substantially influenced by altering the component ratio given in the experimental section. Taking advantage of the heterostructure constructed by ternary components, proper matching behavior, as well as the induced intensified interfacial polarization, were achieved with Sample B. The RL peaks of the samples all shift toward lower frequency bands with increasing thickness—a phenomenon that can be explained by the formula f = c/2πdμ′′. Therefore, the location of the RL peaks can be adjusted for certain applications with different frequencies by manipulating the thickness of the composites. 

**Figure 11:**
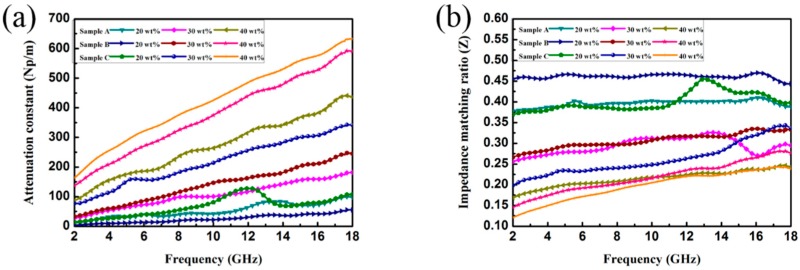
(**a**) Attenuation constant and (**b**) impedance matching ratio for the as-prepared samples containing different loading ratio.

Two key factors should be taken into consideration when designing high-performance EA materials. One is the impedance match and the other is the attenuation capability. The first one requires that the values of permeability and permittivity are roughly equal, which allows the EM wave to enter the materials as much as possible. The second one requires strong EM propagation loss in the interior of the materials. The impedance matching ratio Z, calculated by Z = Z_in_/Z_0_ (Z_in_ is normalized input impedance and Z_0_ is the impedance of free space), can reveal the degree of impedance matching. Actually, when |Z_in_/Z_0_| = 1, the waves can be ensured to enter the absorbents instead of reflecting back to the air, and the impedance matching is optimized. The EM wave attenuation inside the absorber is another important factor for the absorber, and the attenuation constant α of the as-prepared samples is calculated according to the following equation:

(3) $$\alpha =\frac{\sqrt{2}\pi f}{c}\times \sqrt{(\mu^{\prime \prime }\varepsilon^{\prime \prime }-\mu^{\prime }\varepsilon^{\prime })+\sqrt{{\left(\mu^{\prime \prime }\varepsilon^{\prime \prime }-\mu^{\prime }\varepsilon^{\prime }\right)}^{2}+{\left(\mu^{\prime }\varepsilon^{\prime \prime }+\mu^{\prime \prime }\varepsilon^{\prime }\right)}^{2}}},$$

where c is the velocity of light in a vacuum. Figure 11a,b show the frequency dependence of the attenuation constant α, as well as the impedance matching ratio Z for the as-prepared samples with 20 wt.%, 30 wt.%, and 40 wt.%. It should be noted that the values of both Z and α for sample B are located in the middle position among these three samples. As mentioned earlier, impedance matching and attenuation capability should be taken into consideration simultaneously, not their unilateral superior performances. Therefore, the moderate values of Z and α can explain the enhanced EA performance for sample B. Apart from the suitable impedance matching and high attenuation ability of Sample B, its enhanced EA performance can also be attributed to its unique heterostructure formed by ternary components.

For the purpose of strength, camouflage of military equipment, and protecting the health of organisms, EM wave absorbing materials have received lots of attention and are widely studied. In addition to a strong absorption intensity and wide effective absorption bandwidth, materials that are lightweight, thermally stable, and antioxidative are highly desirable. In this study, we fabricated core–shell structured NiS_2_@MoS_2_ nanospheres anchored on rGO nanosheets (NiS_2_@MoS_2_/rGO) by a simple two-step hydrothermal method. The combination ratio was adjusted in order to realize appropriate impedance matching. The EM parameters and the absorption performance were investigated in detail. A composite loaded with 30 wt.% of the sample can achieve a minimum RL value of −29.75 dB, and the effective bandwidth (RL value less than −10 dB) can cover between 4.95 GHz and 18.00 GHz (13.05 GHz) with a thickness ranging from 1.5 mm to 4.0 mm. This study proves that the generated significant interfacial polarization and synergetic interaction between the components can result in NiS_2_@MoS_2_/rGO composites with enhanced EM absorption performance.

## 4. Conclusions

In summary, core–shell structured NiS_2_@MoS_2_ nanospheres anchored on rGO nanosheets (NiS_2_@MoS_2_/rGO) were successfully synthesized by a simple two-step hydrothermal method. As the permittivity varies, the as-prepared NiS_2_@MoS_2_/rGO composites made with the reagent ratio of sample B demonstrate the best EM wave absorbing performance, because various dielectric loss and magnetic loss mechanisms of the composites lead to optimal impedance matching. A composite loaded with 30 wt.% of the sample can achieve the minimum RL value of −29.75 dB and the effective bandwidth (RL value less than −10 dB) can cover from 4.95 GHz to 18.00 GHz (13.05 GHz) with a thickness ranging from 1.5 mm to 4.0 mm. These results indicate that the obtained NiS_2_@MoS_2_/rGO composites have a good EM wave absorbing performance with a wide absorbing frequency, strong absorption, good compatibility, and low density.

## Figures and Tables

**Table 1:** The detailed reaction conditions for preparing NiS_2_@MoS2/reduced graphene oxide (rGO) composites.

Samples	rGO (mg)	NiS_2_ (mg)	(NH_4_)_6_Mo_7_O_24_·2H_2_O (mg)	CH_4_N_2_S (mg)
Sample A	50	50	39	105
Sample B	50	100	39	105
Sample C	50	100	78	210

## Supplementary Materials

The following are available online at http://www.mdpi.com/2079-4991/9/2/292/s1: Figure S1, SEM images of rGO; Figure S2, the energy dispersive X-ray spectrometry (EDS) of the NiS_2_@MoS_2_/rGO composites.
