# Supplementary material for: NiS2@MoS2 Nanospheres Anchored on Reduced Graphene Oxide: A Novel Ternary Heterostructure with Enhanced Electromagnetic Absorption Property
Source: Nanomaterials (Basel). 2019 Feb 19;9(2):292. doi: 10.3390/nano9020292 (PMC6410035; doi:10.3390/nano9020292)
Supplement: Supplementary file 1 [file nanomaterials-09-00292-s001.zip › Supplementary Materials.doc]

**Figure S1.** SEM images of rGO (a).

**Figure S2.** The Energy dispersive X-ray Spectrometry (EDS) of the NiS_2_@MoS_2_/rGO composites (a).
